# A single mutation results in diploid gamete formation and parthenogenesis in a *Drosophila yemanuclein-alpha *meiosis I defective mutant

**DOI:** 10.1186/1471-2156-11-104

**Published:** 2010-11-16

**Authors:** Régis E Meyer, Michèle Delaage, Roland Rosset, Michèle Capri, Ounissa Aït-Ahmed

**Affiliations:** 1 Institut de Génétique Humaine (IGH), Unité Propre de Recherche 1142, Centre National de la Recherche Scientifique (CNRS), 141 Rue de la Cardonille, 34396 Montpellier cedex 5, France; 2 Previous address: IBDML, Campus de Luminy Case 907, 13288 Marseille, Cedex 09, France; 3 Previous address: Department of Biological Sciences, Stanford University, Stanford, CA94305, USA; 4 Current Address: Cell Cycle and Cancer Biology Oklahoma Medical Research Foundation 825 N.E. 13th Street Oklahoma City, Oklahoma 73104 USA

## Abstract

**Background:**

Sexual reproduction relies on two key events: formation of cells with a haploid genome (the gametes) and restoration of diploidy after fertilization. Therefore the underlying mechanisms must have been evolutionary linked and there is a need for evidence that could support such a model.

**Results:**

We describe the identification and the characterization of *yem^1^*, the first *yem-alpha *mutant allele (V478E), which to some extent affects diploidy reduction and its restoration. Yem-alpha is a member of the Ubinuclein/HPC2 family of proteins that have recently been implicated in playing roles in chromatin remodeling in concert with HIRA histone chaperone. The *yem^1 ^*mutant females exhibited disrupted chromosome behavior in the first meiotic division and produced very low numbers of viable progeny. Unexpectedly these progeny did not display paternal chromosome markers, suggesting that they developed from diploid gametes that underwent gynogenesis, a form of parthenogenesis that requires fertilization.

**Conclusions:**

We focus here on the analysis of the meiotic defects exhibited by *yem^1 ^*oocytes that could account for the formation of diploid gametes. Our results suggest that *yem^1 ^*affects chromosome segregation presumably by affecting kinetochores function in the first meiotic division.

This work paves the way to further investigations on the evolution of the mechanisms that support sexual reproduction.

## Background

Sexual reproduction relies on two key mechanisms: meiosis that yields haploidy and syngamy that restores diploidy. Meiosis is, with mitosis, one of the two strategies used by eukaryotes to propagate their genome. Despite the similarities between these processes, the main differences account for ability of meiosis to result in the formation of gametes with a haploid genome whereas mitosis results in a faithful transmission of the diploid genome to the daughter cells ([1] and references therein). Several differences stand out when comparing meiosis and mitosis. First, in meiosis, a single round of DNA replication is followed by two successive divisions: meiosis I that segregates the chromosomes with homologous centromeres (reductional division) and meiosis II that segregates the sister centromeres (equational division). The migration of sister chromatids to the same pole, which is unique to meiosis I, is accomplished through meiosis-specific modifications to sister kinetochores such that they display an attachment to microtubules emanating from the same pole, and through protection of sister chromatid cohesion near the centromeres, which keeps the sisters together until meiosis II. Meiosis II, in contrast, requires bipolar attachment of sister kinetochores at metaphase II and complete removal of centromeric cohesion to allow progression to anaphase II and equational segregation of sister chromatids ([2] and references therein).

Another significant difference is that recombination during prophase I between non-sister chromatids links the homologues in a structure termed a bivalent. This linkage allows the homologous partners to attach to the meiotic spindle in a manner that will result in their disjunction at anaphase I. Recombination may be absent, as in *Drosophila *males [3], but whenever it occurs as a normal programmed process, it is included in the strategy that ensures accurate chromosome disjunction at meiosis I. Interestingly, *Drosophila *females have to deal with the necessity to segregate both exchange (chiasmate) and non exchange (achiasmate) chromosomes [4].

These events have been well characterized in yeast. However, in spite of their universality they may be supported by different strategies and protein sequences in different organisms. Rec8, an Scc1/Rad21 meiosis-specific paralogue allows the two-step removal of cohesin, along the chromosome arms in meiosis I and at the centromeres in meiosis II, in both *Saccharomyces cerevisiae *and *Schizosaccharomyces pombe*. The meiosis I-specific monopolar orientation of sister kinetochores relies on different protein complexes, involving Rec8 and Moa1 for *S. pombe*, and the monopolin protein complex for *S. cerevisiae*. Rec8 that is also required for meiotic recombination is loaded on the chromosomes at pre-meiotic S-phase whereas monopolin is loaded during meiotic prophase once recombination is completed [5-9].

Interestingly, neither Rec8 nor monopolin are found in *Drosophila*. Nonetheless in *Drosophila*, the meiosis-specific functions such as specific cohesion and mono-orientation of sister chromatids must be supported by meiosis-specific proteins. Earlier screens for meiotic mutants in *Drosophila *were generally based on a search for mutations that affect recombination and/or chromosome disjunction [10-12]. These genes have been analyzed in the last decades (for review see [13] and references therein). Strikingly, in *Drosophila*, the factors that are critical for monopolar orientation of sister kinetochores in meiosis I have remained elusive. This is in part likely due to the bias of phenotypic screens, which have often depended upon the production of viable adult progeny from mutant females.

We identified *yem-alpha *in an alternative screen for genes specifically expressed in the female germ line. It encodes an oocyte specific DNA binding protein [14]. Very recently Yem-alpha/Ubinuclein/HPC2 family of proteins have been shown to be involved in the HIRA mediated chromatin remodeling complexes in Humans, Yeasts and Drosophila [15-17]. But because of the paucity of genetic tools at the *yem-alpha *locus its biological function remained elusive up to the present work.

Here we report the first mutant allele of *yem-alpha *(*yem^1^*) obtained in a screen for female sterile mutations, and experiments performed using this allele to explore the meiotic role of *yem-alpha*. Yem-alpha was found to colocalize with CID, the *Drosophila *histone H3 variant specific for the kinetochore. The *yem^1 ^*point mutation (V478E) affects chromosome behavior at meiosis I. At metaphase I *yem^1 ^*oocytes display defects in aligning on the meiotic spindle suggesting kinetochore dysfunction. In a recombination defective context, the *yem^1 ^*oocytes undergo precocious anaphase and female sterility is partially suppressed. This results in the development of parthenogenetic exceptional progeny. The analysis of their X chromosome markers shows that these progeny are formed from diploid eggs that inherited the two maternal homologues. The cytological and the genetic data combined suggest the possibility that these eggs are diploid as a result of kinetochore defects induced by *yem^1 ^*mutation. Indeed kinetochore dysfunction may result in a single equational division.

## Results

### Identification and characterization of the first yem-alpha mutant allele (yem^1^)

*Drosophila yem-alpha *was identified in a search for genes that are differentially expressed in the female germ line [14,18,19]. We showed that Yem-alpha is a DNA binding protein specific for the oocyte nucleus [14]. Lack of genetic tools at the *yem-alpha *locus was a major pitfall in the identification of its biological function. Accordingly, an EMS mutagenesis strategy was used to isolate mutations in the *yem-alpha *locus (Additional file 1). Mutations that exhibited female sterile phenoypes when combined with a synthetic deletion that removes *yem-alpha *[20] were screened to identify any that could be rescued by a *yem-alpha *expressing transgene. A single *yem-alpha *mutant allele was identified named *yem^1 ^*(Additional file 1). Females homozygous for *yem^1 ^*were sterile, and, as expected, males were not affected by the mutation. A majority of the mutant eggs (70%) entered the mitotic cycles but the embryos failed to hatch (data not shown). Because in *Drosophila *the formation of the first mitotic spindle of the zygote is dependent on the centrosomes provided by the sperm cell [21]*yem^1 ^*female sterile phenotype cannot be attributed to fertilization defects.

**Figure 1:**
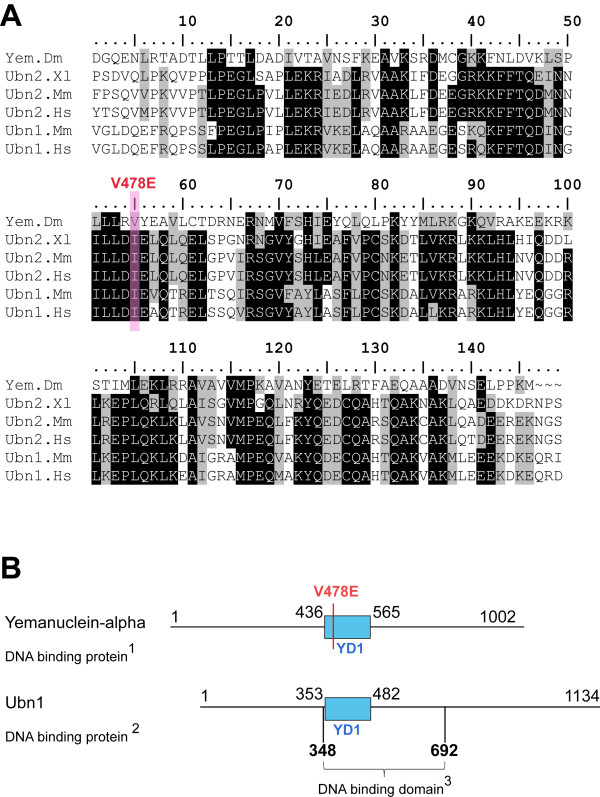
**Sequence conservation of the domain that contains V478E yem^1 ^mutation(YD1)**. The V478 containing domain was subjected to a Blast search and Yem-alpha like sequences were aligned with CLUSTAL-W (Panel A). The alignment was optimized with BioEdit. Grey and black shadings highlight similar and identical residues respectively using a 60% consensus. To the exception of Yem-alpha and Ubn1 the other sequences are putative proteins, Yem-alpha being the archetypal sequence published long before the human orthologue [14,22]. Accession numbers are given in Additional file 1, Table S1. Panel B is a schematic representation of the YD1 domain of Ubn1 and Yem-alpha. Yem-alpha DNA binding properties have been determined experimentally [14]. The nucleotide sequence of the Ubn1 DNA binding domain was previously reported as "VT4 cDNA" (Additional file 1, Table S1).

In order to gain insight into the molecular basis of the mutant phenotype, we sequenced the mutant allele and also the wild type allele from the isogenized chromosome used in the mutagenic screen (Additional file 1). A single difference was found that translates into a missense mutation resulting in replacement of Valine 478 by Glutamic acid (V478E). Sequence alignments between Yem-alpha and vertebrate related sequences (*Homo sapiens, Xenopus laevis, Mus musculus*) show that V478 falls in one of the three Yem-alpha conserved domains, which spans amino-acid 436 to amino acid 565. Val478 is located within a highly hydrophobic hexapeptide. It is noteworthy that hydrophobic cores are essential for protein structure/function, which is in good agreement with the high conservation of the hydrophobic positions (Figure 1A and Additional file 1, Table S1). Interestingly, the V478E mutation falls within a Ubn1 region implicated in DNA binding [22]. The domain that bears V478 was named YD1 for Yemanuclein Domain 1 (Figure 1, panel B).

### The V478E yem^1 ^mutation affects meiosis I

**Figure 2:**
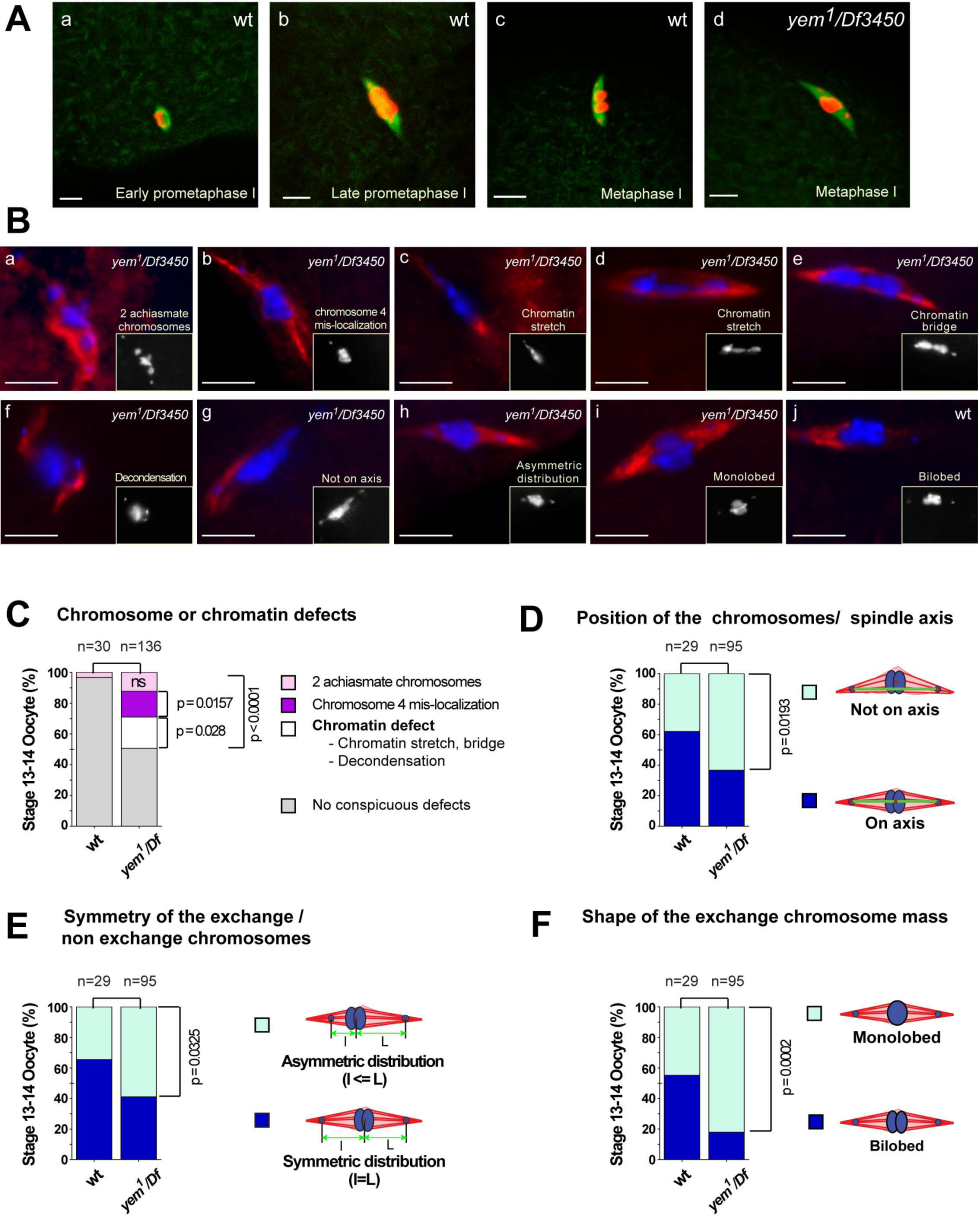
**Chromosome defects of the yem^1^/Df3450 mature oocytes**. The oocytes were treated as indicated in the experimental procedures prior to proceeding with the staining of the meiotic spindles. A - Projections of confocal sections showing meiotic spindles. The microtubules were revealed with anti-tubulin antibodies (green) and the condensed chromatin with anti-Phospho-histone H3 antibodies (red). The Drosophila oocyte spindle is acentriolar and nucleates at the chromatin mass as shown in (a). Late prometaphase is characterized by the non exchange 4^th ^chromosomes budding over the masses of the chiasmate chromosomes (b). In a fully mature stage 14 oocyte the exchange chromosomes are arrested at metaphase I whereas the achiasmate 4^th ^chromosomes undergo anaphase I (c). Note the irregular chromatin mass of the *yem^1 ^*exchange chromosomes (d). Scale: the bar represents 5 μm. B - Conventional epifluorescence pictures. Mutant and wild type oocytes were stained for DNA (DAPI) and the spindle with anti alpha-tubulin antibody (red). This gallery displays various phenotypic classes. Scale bar equals 5 μm. C-F - Histograms representing the ratio of the different phenotypic classes analyzed in this work. The first histogram represents the analysis of the conspicuous defects observed on a large number (n = 136) of oocytes. A detailed analysis was performed on a 95 spindles fraction. (n) Represents the number of oocytes scored. *Df *represents *Df3450*. To determine whether quantitative differences between two classes are significant, we used the two-tailed distribution of Fischer'exact test http://www.graphpad.com/quickcalcs/. The p-value states the probability for the null-hypothesis (i.e, that the differences are due to sampling variations).

Because of Yem-alpha specific localization to the oocyte nucleus, we hypothesized a meiotic function [14,23]. Our hypothesis was supported by recent investigations that allowed us to show Yem-alpha association to the synaptonemal complex and its role in meiotic recombination (Meyer et al, in preparation). However the first conspicuous cytological defects are not observed before the stage 14 oocyte. At this stage meiosis is considered to be arrested at metaphase I. Although a recent publication proposes a new definition for the meiotic stages in the *Drosophila *oocyte [24], the staging used here relies on the one that has been widely used by the community [4] according which metaphase I is reached when the spindle is fully elongated. At this stage, the chromatin is arranged as a characteristic symmetrical structure; with the exchange chromosomes positioned as two adjacent masses at the spindle mid-zone and the non-exchange 4^th ^chromosomes positioned between the central mass and the poles (Figure 2A). The spindle looks atypically tapered with the highest microtubule concentration at the vicinity of the chromatin masses [4]. This configuration is a consequence of an asterless spindle that nucleates at the chromatin at early prometaphase. The mutant oocytes had a symmetry that was often disrupted, exhibiting irregular monolobed chromatin masses. Other defects may be observed as shown in Figure 2B. In this experiment the stage 14 oocytes were labeled for DNA (DAPI) and for the microtubules (anti alpha-tubulin). Because even in wild type oocytes the spindles do not always display regular shapes we estimated the extent to which the irregularities shown in Figure 2B were real defects due to the V478E mutation. This analysis and the statistical p-values that result from the application of the Fischer exact test are shown in Figures 2C,D,E,F.

We first performed a crude analysis on a large number of mutant spindles (136) to assess the frequency of conspicuous defects in chromosome behavior (mis-localization of achiasmate 4th chromosomes on the spindle, extra achiasmate chromosomes) and in chromatin organization (stretches, bridges, decondensation). It appears from this analysis that the V478E mutation results in significant conspicuous defects for half of the oocytes analyzed in this study (p-value < 0.00001). Significantly higher levels of mis-localization of chromosome 4 on the spindle (p-value = 0.0157) and chromatin defects (p-value = 0.028) were scored in the mutant oocytes. We then compared wild type and mutant meiosis I spindles for the following parameters: symmetry of the congressed chromosome masses relative to the spindle axis (Figure 2D) and to the position of the achiasmate 4th chromosomes (Figure 2E). The position of the non-exchange chromosomes was chosen as a reference instead of the spindle poles as the microtubules may be very faint at the poles. The shape of the masses of the congressed exchange chromosomes was the last parameter to be considered in this analysis (Figure 2F). These 3 parameters could be analyzed on 95 specimens out of the 136 analyzed in Figure 2C as the chromatin of the other 41 specimens was too heavily disorganized.

It appears from this analysis (Figures 2D,E,F) that all the symmetries considered were significantly affected by the V478E mutation: position of the chromosomes on the spindle axis (p = 0.0193), symmetry of the exchange versus non-exchange chromosomes (p = 0.0325) and shape of the exchange chromosome mass (p = 0.0002).

Altogether these data, added to the DNA binding properties of Yem-alpha [14], suggest that *yem^1 ^*mutation affects the chromatin in ways that disrupt organization of the chromosomes on the meiotic spindle. The shape of the chromosome masses (monolobed versus bilobed) could also be due to aberrant number and/or distribution of crossovers.

### In a recombination defective background yem^1 ^chromosomes fail to perform the two meiotic divisions within the oocyte

Then we wanted to analyze whether the alignment defects of *yem^1 ^*chromosomes affected meiosis progression. We used genetic backgrounds where the chromosomes were relieved from crossovers, which in *Drosophila *results in premature meiosis progression within the oocyte.

*Drosophila *meiosis is arrested at metaphase I at the end of oogenesis, in the mature oocyte (stage 14). In normal conditions, meiosis resumption is triggered by ovulation and is completed within 20 min after egg laying [25]. The metaphase I arrest in the *Drosophila *stage 14 oocytes results from the tension generated by the chiasmata that link the arms of the homologues and the kinetochores [26]. Consequently, as recombination defective mutants do not form chiasmata, they fail to pause at metaphase I and undergo precocious anaphase [27].

**Figure 3:**
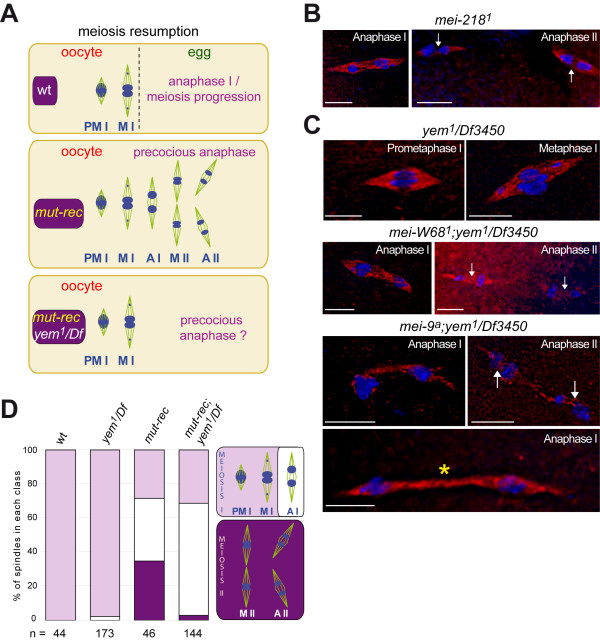
**yem^1 ^chromosomes bypass the meiotic arrest in a recombination defective background but fail to undergo the two divisions within the oocyte**. A - Schematic representation of meiosis progression in stage 14 oocytes. In a wild type oocyte, the meiotic cycle pauses at metaphase I as a result of the tension generated between chiasmata and kinetochores. Meiosis resumption is triggered by ovulation. Recombination defective mutants (*mut-rec*) do not experience metaphase I arrest in the oocyte; their chromosomes undergo precocious anaphase as does achiasmate 4th chromosome. (B, C) Gallery of pictures showing oocytes with various recombination defective genotypes stained for DNA (DAPI) and the spindle (anti alpha-tubulin antibody). (B) Precocious anaphase (left) and anaphase II (right) spindles in *mei-218^1 ^*oocytes. (C) Prometaphase and metaphase I *yem^1^/Df3450 *oocytes (upper panel). Precocious anaphase I and exceptional anaphase II (arrow) in *mei-W68^1^; yem^1^*/*Df3450 *and *mei-9^a^; yem^1^/Df3450 *oocytes (lower panel). Note that the mutant chromosomes are not equally partitioned on the spindle. The last picture (*) shows a highly elongated double mutant spindle. Scale: the bar represents 5 μm. D - Histogram representing the ratio of the 3 classes of meiotic figures scored for 4 different genotypes. (n) Represents total number of oocytes scored for each genotype. The 3 meiotic classes considered here are: prometaphase, metaphase I (light purple), anaphase I (white) and meiosis II (dark purple). In a *mut-rec *background (here *mei-W68^1 ^*and *mei-9^a^*), *yem^1 ^*mutants bypass the metaphase I arrest. In contrast to simple recombination defective mutants (*mut-rec*), meiosis II spindles are hardly observed in *mut-rec; yem^1^*/*Df3450 *oocytes. Obviously these mutant chromosomes were not able to support the two meiotic divisions within the oocyte. Abbreviations: PMI (prometaphase I); MI (metaphase I); AI (anaphase I); MII (metaphase II); AII (anaphase II).

Various mutations may result in defective recombination and consequently suppress (or reduce) chiasma formation such as mutations that affect formation or maintenance of the synaptonemal complex (*c(3)G*, *c(2)M*, *ord*), double strand break formation or other early steps of the meiotic recombination process (*mei-W68 *or *mei-218 *and *mei-217*). The *mei-9 *mutation acts later by preventing crossover formation [13]. These mutations are summarized in Additional file 1, Table S2. Not only do the recombination defective oocytes fail to arrest at metaphase I but they may also undergo (and complete) the second round of the meiotic cycle [27]. Consequently, meiosis II spindles may be scored in such oocytes (Figures 3A and 3B).

To analyze the effect of the *yem^1 ^*mutation in the absence of chiasma, we generated *yem^1^/Df3450 *females that are also mutant for one of the genes required in the recombination process (conveniently called *mut-rec*). The *mut-rec *mutants used in this work are described in Additional file 1, Table S2. The *mut-rec; yem^1^/Df3450 *stage 14 oocytes were stained for the DNA and the spindle (Figure 3C). As shown in Figure 3C in a recombination defective context, regardless of the *mut-rec *mutant used for the experiment, *yem^1 ^*oocytes undergo precocious anaphase. However, while a significant number of *mut-rec *oocytes display two spindles, this is rarely observed in the *mut-rec; yem^1^/Df3450 *oocytes (Figure 3C). A quantitative analysis supporting these observations is shown in Figure 3D. The meiotic stages were scored in 4 different genetic contexts: wild type, *yem^1^/Df3450*, *mut-rec *and *mut-rec; yem^1^/Df3450*. Prometaphase I and metaphase I spindles were considered here as a single class; anaphase I and meiosis II spindles were scored separately as schematically represented in panel D (right). Approximately 30% of the *mut-rec *oocytes reached meiosis II. These results are in perfect agreement with published data [27]. In contrast, less than 3% of the *mut-rec; yem^1^/Df3450 *mutant oocytes score typical meiosis II spindles in spite of a similar ratio of oocytes that undergo precocious resumption of the meiotic cycle (Figure 3D).

In conclusion, in a recombination defective context, although they were able to perform precocious anaphase, *yem^1 ^*chromosomes were not competent to perform the two rounds of the meiotic cycle within the oocyte. Interestingly the anaphase spindles of the double mutants are often highly stretched, suggesting that anaphase I is delayed (Figure 3C, yellow asterisk). This delay might result for some eggs in the skipping of the first segregation step leading to the formation of diploid eggs. However the failure to perform the two meiotic cycles could be temporary for most eggs.

### Recombination defective mutations partially rescue the sterility of the yem^1^/Df3450 females but their exceptional offspring are parthenogenetic

**Figure 4:**
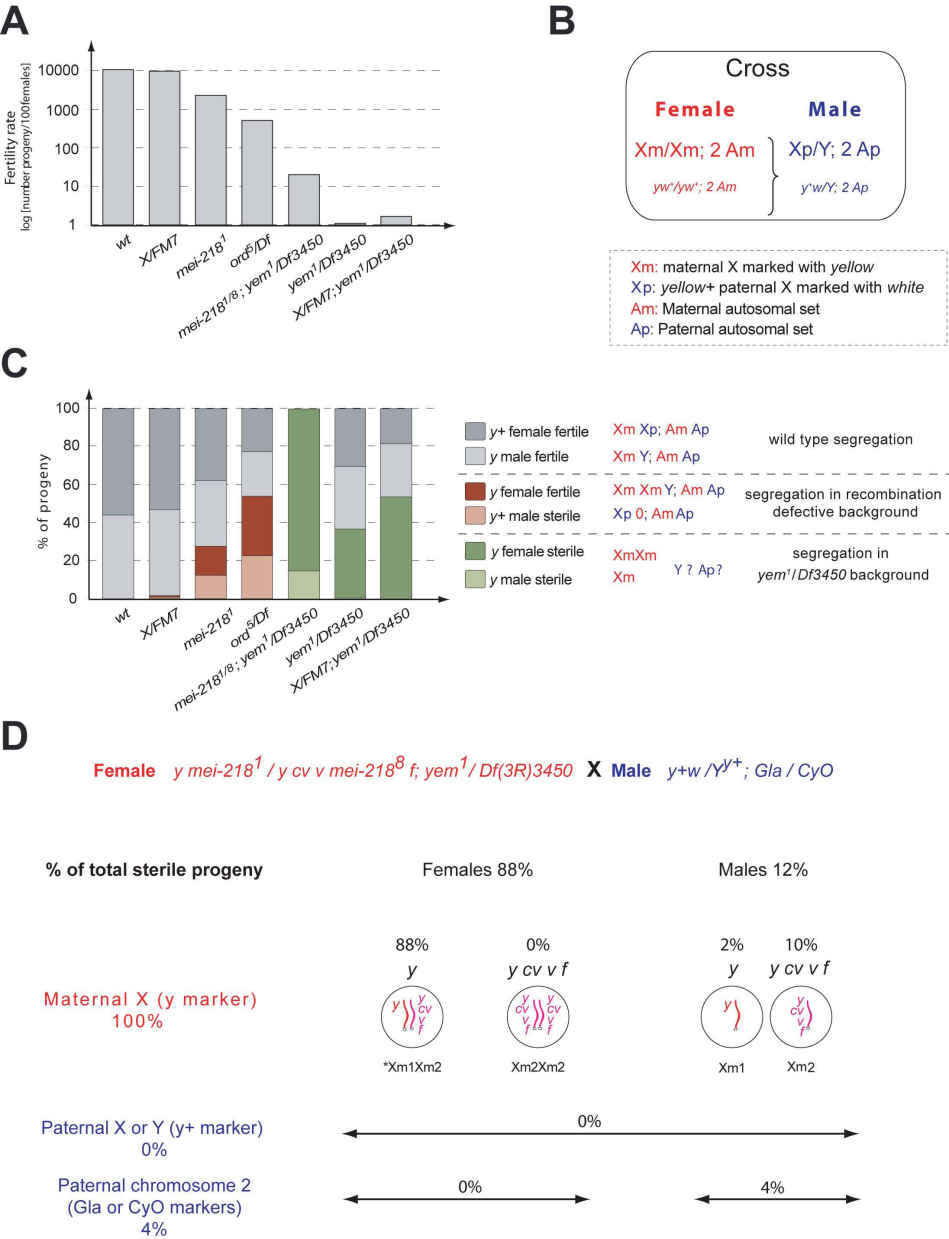
**Fertility rate and chromosome segregation in various genotypes**. *Fertility rate *was expressed as progeny number for 100 mothers (A). The genotypes were either wt, with or without achiasmate X (*X/FM7*), mut-rec (*mei-218^1^*, *ord^5^/Df*) or mutant for *yem-alpha *(*yem^1^/Df3450*, *X/FM7; yem^1^/Df3450 *and *mei-218^1/8^; yem^1^/Df3450*). *Chromosome segregation *(B, C, D). In (B), the cross on which we based the analysis shown in (C). The bar graph (C) represents the % of total progeny falling in the 6 classes scored as indicated in panel C. Histogram drawing was after data contained in Additional file 1, Table S3. In grey, progeny obtained from eggs that underwent normal segregation. In brown, progeny with X missegregation as observed in *mut-rec *mutants; sterile males are X0 (*y^+^w*). In green, *y *sterile progeny specifically recovered in *yem^1 ^*background. Sterile males have no paternal markers (*yw^+^*). (D) To ask how paternal chromosomes are transmitted and why the progeny in green are sterile we performed a cross between *mei-218^1/8^*; *yem^1^/Df3450 *females and males marked on both the sex chromosomes and the autosomes. Paternal X (Xp) is *y*^+^*w *as above and the Y chromosome bears a translocated X-linked *y+ *locus (Y^y+^). Chromosomes II have *Gla *or *CyO *dominant markers. 96% of the *y *sterile progeny had no paternal markers. Therefore the class represented in green in panel C represents essentially parthenogenetic females and some Xm0 males. One Xm chromosome bears a specific set of recessive markers (*cv v f*), which allows inferring the type of segregation (Xm1, Xm2). * As no *y cv v f */*y cv v f *progeny were recovered, we infer that the females developed from diploid eggs that were *y*/*y cv v f*. More explanations are provided in the text and also in Additional file 1, Table S3.

In order to further investigate the effect of the recombination defective background, we were interested in analyzing its capacity to restore fertility in the *yem^1^/Df3450 *females, our ultimate goal being to gain insight into chromosome segregation in this mutant background. Interestingly a small but significant proportion of the eggs produced by the *mut-rec; yem^1^/Df3450 *females were able to support development of progeny up to adulthood. The ratio of the fertility rate of the mutant females/the fertility rate of the wild type is highest (0.2%) with the *mei-218^1/8 ^*allelic combination (Figure 4A and Additional file 1, Table S3). Because of their low fertility, crosses were carried out with a large number of *mut-rec; yem^1^/Df3450 *females (Additional file 1, Table S3). Following phenotypic markers in the exceptional adult progeny assessed the X chromosome segregation (Figure 4B). The maternal X chromosome (Xm) was marked with *yellow *(*yw^+^/yw^+^*) while paternal X (Xp) was marked with *white *(*y^+^w*). The genetic analysis is schematically shown in Figure 4C and Additional file 1, Table S3. Some features stand out when analyzing these data. In a *mut-rec*; *yem^1^/Df3450 *genetic background, the progeny were *yellow *which is consistent with a non-disjunction in the mother. Strikingly, progeny from *mut-rec*; *yem^1^/Df3450 *were sterile when tested in regular conditions. If these *yellow *female progeny developed from eggs that were disomic for the X chromosome as a result of classical non-disjunction, then one might expect the *mut-rec*; *yem^1 ^*females to produce an equal number of nullo-X eggs. Such eggs, when fertilized by normal sperm carrying an X chromosome should give rise to *y^+^w *males (Xp0) that are sterile, as in *Drosophila *the Y chromosome is necessary for male fertility (although not required for sex determination). The absence of these progeny suggests that the *y *female progeny do not arise from simple non-disjunction and instead points to a different kind of meiotic outcome, defining *yem^1 ^*as a new class of mutants.

To determine whether the exceptional progeny in the above experiment were attributable to the *yem^1 ^*mutation and not the *mut-rec *mutant background, we searched for these same classes exceptional progeny among the very rare progeny produced by recombination proficient *yem^1^/Df3450 *females. Some progeny may be recovered when crosses are carried out with a large number of females (Additional file 1, Table S3). As shown in Figure 4A, the fertility rate drops to 0.01% as compared to the wild type; it approaches 0.02% in the presence of the *FM7 *achiasmate X chromosome (Figure 4A and Additional file 1, Table S3). The unpredicted *yellow *sterile progeny described above were also recovered from these females. In a recombination proficient background, these progeny represent less than 40% of total progeny. The ratio reaches nearly 60% in the presence of an achiasmate X chromosome (it is 100% in a *mei-218^1/8 ^*recombination defective background). Therefore, reducing recombination partially restores fertility and increases the ratio of the *yellow *and sterile progeny.

At this point two observations are still puzzling: the absence of paternal X markers (*y^+^w*) in the exceptional progeny of *mut-rec; yem^1^/Df3450 *females and the genetic basis for their sterility. XXY individuals may be recovered when an XmXm female pronucleus undergoes syngamy with a male pronucleus that has a Y chromosome. Such individuals would be phenotypically *yellow *but they would be fertile females provided they received normal maternal (Am) and paternal (Ap) sets of autosomes. Therefore, there was a possibility that the autosome sets might not be AmAp in these *yellow *and sterile progeny. To test this possibility, we performed a cross in which both the sex chromosomes (X and Y) and the paternal autosomes were marked. Chromosomes II were dominantly marked with *Gla *and *CyO *and we used a Y chromosome with a translocation of the X-linked *yellow+ *locus (Y^*y+*^). The genetic cross is shown in Figure 4D. The progeny would be expected to express the *yellow+ *phenotype if the paternal sex chromosomes were transmitted. But the observed progeny did not (Figure 4C). These progeny were 88% *yellow *females (XmXm) and 12% *yellow *males (presumably Xm). 96% of these *yellow *progeny received no paternal autosomes (Figure 4D and Additional file 1, Table S3). The absence in these flies of chromosomes from the male parent demonstrates they developed parthenogenetically from diploid eggs produced by the *mut-rec*; *yem^1^/Df3450 *females (*mut-rec *being here *mei-218*). Accordingly these progeny were mostly female. The presence of parthenogenetic males may be accounted for by the presence of diploid eggs that are monosomic for the X chromosome. Indeed the flies that receive a maternal X and no paternal sex chromosome (Xm0p) are expected to be sterile males.

This finding sheds light on the sterility of the *yellow *progeny. These flies inherited the *yem^1 ^*and *Df3450 *mutant third chromosomes. In the absence of a paternal wild type allele of *yem-alpha*, this combination confers sterility in females (as tested in standard experimental conditions). The presence or absence of a *yem-alpha *wild type allele was assessed by genotyping the *yellow *individuals. A PCR fragment that encompasses YD1 domain was sequenced. As expected, only the V478E allele was present in these flies. In contrast, the sterility of the *yellow *males (12% of progeny) is best explained by the lack of a Y chromosome.

4% of the *yellow *sterile individuals (about one-third of the males that comprised 12% of the total progeny) had the paternal autosomal markers (*Gla *or *CyO*) (Figure 4D and Additional file 1, Table S3). These males presumably lost the paternal sex chromosome (X or Y) at the first mitotic division as no mosaicism was observed. Such individuals reveal an abnormal rate of chromosome instability even when normal sets of chromosomes were provided to the zygote (for details see Figure 4 and Additional file 1, Table S3). The cause of this instability is unclear but it is certainly related to the *yem^1 ^*mutant background.

### The diploid mut-rec; yem^1^/Df3450 eggs have homologous sets of maternal chromosomes

Because of the meiotic defects displayed by the *mut-rec; yem^1^/Df3450 *females, we assumed that the formation of the diploid gametes resulted from defective chromosome segregation. In the crosses shown in Figure 4D one of the two maternal X chromosomes bearing specific recessive markers (*cv v f*) it was possible to analyze the type of segregation that resulted in the formation of the diploid eggs. The analysis is schematically shown in Figure 4D and in detail in Additional file 1, Table S3. None of the *yellow *exceptional female progeny expressed the *cv v f *markers. These markers being perfectly expressed in the parthenogenetic X0 males, this chromosome would have been scored in *cv v f *homozygous females.

Similar results are obtained in analyses performed with different recombination defective backgrounds but the extent to which the parthenogenetic offspring were recovered depends on the recombination defective mutation used in combination with *yem^1 ^*(Additional file 1, Table S3). This underscores the importance of the role played by the genetic background (regarding meiotic recombination) in the formation of the diploid eggs. These data are in agreement with a defective meiosis I and suggest the possibility that the exceptional progeny develop from diploid eggs that result from a unique equational division.

### Yem-alpha localizes to the kinetochore

Our data suggest an analogy between the segregation defects described here and those described in yeast mutants that are defective for kinetochore functions [7,28,29]. Indeed the two meiotic cycles rely on a specific organization of the sister kinetochores. The formation of diploid gametes that bear the markers of the two maternal chromosomes could be explained by a failure or block to meiosis I followed by a single meiosis II - like division that separates sister chromatids. In budding yeast, this can occur when cells fail to assemble meiosis-like kinetochores which enables sister kinetochores to attach to the same spindle pole at meiosis I. In these mutants, the kinetochores of sister chromatids attach to opposite poles at meiosis I (rather than waiting until meiosis II). This premature bipolar attachment of the sister kinetochores can block meiosis I (because cohesins keep the sisters from being pulled apart) followed by a division in which mainly sisters disjoin to form elevate levels of diploid spores. If the *yem^1 ^*diploid eggs arise from a similar process in spite of their very low abundance, then Yem-alpha might be predicted to act at kinetochores.

**Figure 5:**
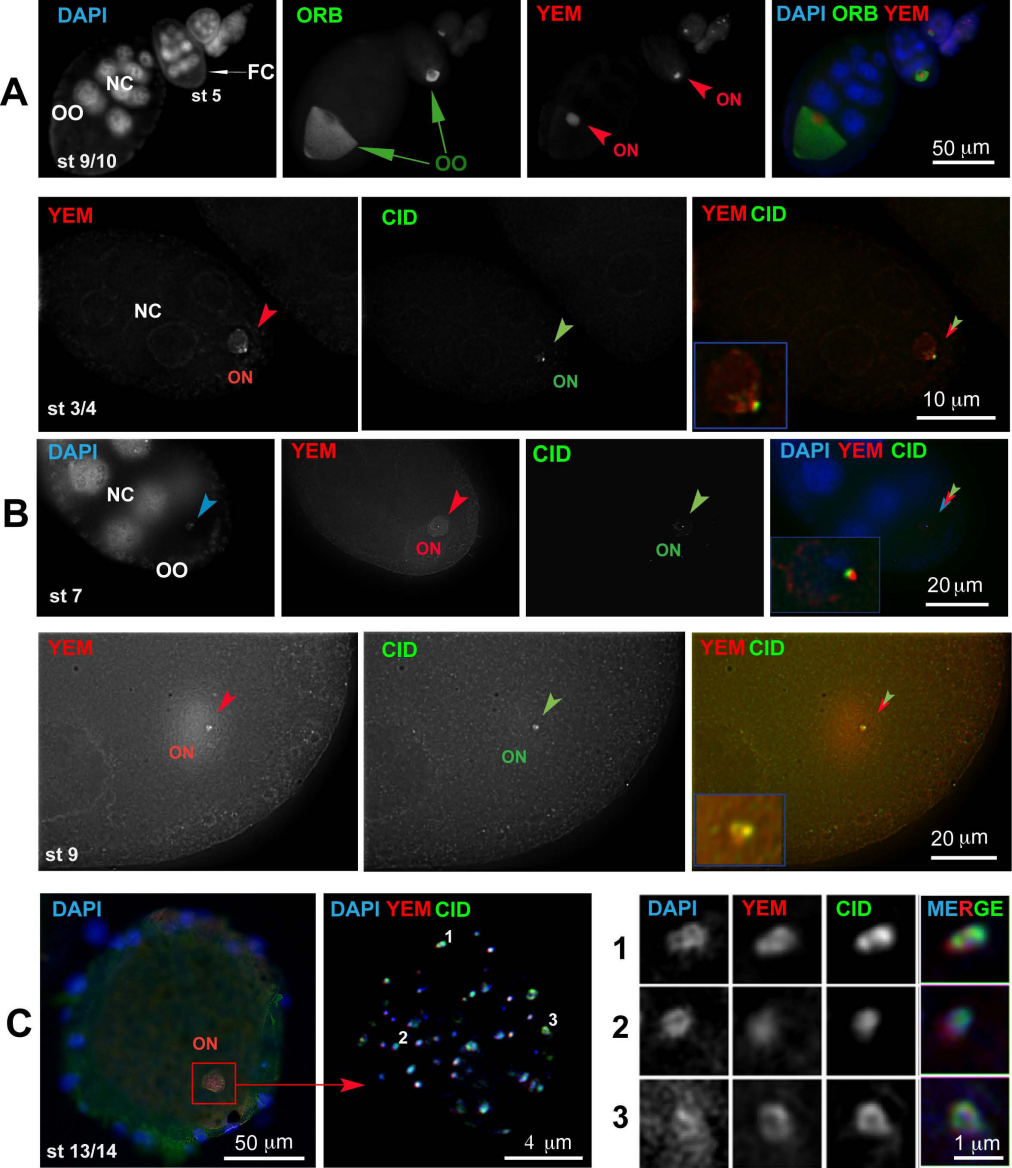
**Immunolocalization of Yem-alpha and centromeric CID on oocyte whole mounts and cryo-sections**. Whole mounts (Panels A and B) and cryosections (panel C) were observed by epifluorescence. To the exception of the pictures of panel (A), all other pictures were deconvolved. A - The panel represents staining of whole mount ovary for DNA (DAPI), Yem-alpha [14,23] and Orb [52]. Yem-alpha is specifically localized to the oocyte nucleus whereas Orb specifically marks the ooplasm [14,52]. FC: follicle cells NC: nurse cells; OO: ooplasm; ON: oocyte nucleus; st: oocyte stage. The arrow points out the oocyte and the arrowhead points out the oocyte nucleus. B - Gallery of whole mount egg chambers of various stages stained as indicated in the Methods section with anti Yem-alpha [14,23] and anti-CID antibody [30]. Yem-alpha is localized within the whole nucleoplasm with the highest intensity colocalizing with the CID signal that specifically marks the centromeric region. The arrowhead points out the oocyte nucleus (ON). C - The panel displays a 40× magnification of a transverse section through the meiotic spindle of a mature oocyte (left), a 160× magnification of the same nucleus (middle) and zooms on 3 representative structures that are numbered 1, 2, 3 (right). These pictures were deconvolved. Yem-alpha staining was performed using a rabbit polyclonal antiserum [14,23]. CID was revealed with a chicken anti-CID antibody [30]. DNA was stained with DAPI.

We addressed this issue by immunostaining for both CID, the centromeric histone H3 variant that marks the inner kinetochore [30], and Yem-alpha using the same polyclonal antibody that specifically stains the oocyte nucleus [14,23]. The results are shown in Figure 5. Yem-alpha staining throughout oogenesis is shown for an ovariole at low magnification (Figure 5A). Yem-alpha is abundant in the nucleoplasm; only a fraction of the protein is associated to the chromosomes (Meyer et al, in preparation). Therefore to assess its centromeric localization observations at higher magnifications and deconvolved pictures were required (Figures 5B,C). For early oogenic stages (up to stage 9), whole mount stainings were conveniently used. As shown in Figure 5B, Yem-alpha and CID colocalize. But because Yem-alpha antigen is destroyed by classical fixation methods, the whole mounts were performed without prior fixation. However, such a method cannot be applied to mature oocytes as fixation is an absolute requirement before egg coverings removal, which is a prerequisite for antibody penetration. To circumvent this difficulty we performed frozen sections as previously described [14]. A transverse section through the spindle of a mature oocyte is shown in Figure 5C. CID staining was consistently found in association with DAPI and Yem-alpha as co-localized dots. However not all the DAPI and Yem-alpha positive dots are also positive for CID. This is in agreement with CID specific localization to the centromere whereas DAPI and Yem-alpha are expected to localize to the chromosome arms. When by chance the section crosses perfectly the sister kinetochores, it is possible to see a pair of dots that are stained for DAPI, CID and Yem-alpha (Figure 5C). This is reminiscent of the typical "side by side" arrangement of the meiotic sister kinetochores [31].

We are aware that the cytological analysis of Yem-alpha on *Drosophila *female meiotic chromosomes approaches its limit. We have analyzed Yem-alpha localization on grasshopper meiotic chromosomes as they provide superior cytology. At metaphase I the staining could be observed at the kinetochores and between the sister chromatids arms, a result that supports our cytological data (Aït-Ahmed and Rufas, unpublished observations).

## Discussion

### The yem-alpha V478E mutation affects female meiosis I

We identified *yem-alpha*, a gene that was overlooked in previous screens for actors of *Drosophila *female meiosis. We reported its molecular characterization in an earlier work [14] and we showed that *yem-alpha *RNA is concentrated in the oocyte throughout meiosis I from the earliest germarial stages [23]. Accordingly Yem-alpha protein was detected in the oocyte nucleus throughout meiosis I (Meyer et al, in preparation). From its specificity for the oocyte nucleus and its affinity for DNA we assumed a role in female meiosis but lack of genetic tools eluded its function for two decades. We report in the present work, the first mutation of *yem-alpha *(*yem^1^*) that highlights some of its functions in *Drosophila *female meiosis I.

### The yem^1^/Df3450 females produce exceptional progeny that are parthenogenetic

Exceptional progeny that develop from eggs laid by *yem^1^/Df3450 *females could be scored only when non-conventional crosses were performed with a high number of mutant females. The analysis of X chromosome segregation reveals two essential features: missegregation and formation of unexpected rare progeny. The chromosomal composition of these progeny suggests the *yem^1 ^*mutation must impact two aspects. First, the presence of two chromosome sets from the mother suggests that in *yem^1 ^*mutants, diploid eggs must form at some frequency. Second, *yem^1 ^*must also somehow disallow the presence of male chromosomes, as the progeny are parthenogenetic. These progeny were recovered from both proficient and recombination defective females. Therefore parthenogenesis can be attributed only to the *yem^1^/Df3450 *genetic background.

*Drosophila melanogaster *seems to be pre-adapted for parthenogenesis. This was recognized more than 20 years ago by Fuyama [32,33]. This Author established a *Drosophila *line called *gyn-F9 *that developed gynogenetically when mated to *ms(3)K81 *sterile males. It is worth noting that *Drosophila melanogaster *requires fertilization, as plasmogamy is a pre-requisite for egg development, the centrosomes being provided by the sperm cell [21]. This type of parthenogenesis is also called gynogenesis. Interestingly, unlike the *yem^1 ^*females, when crossed to fertile males, *gyn-F9 *females are able to produce triploids and intersexes. Therefore syngamy is possible between the diploid *gyn-F9 *female pronucleus and the male pronucleus [32,33]. No triploids were recovered in the exceptional progeny of the *yem^1^/Df3450 *females, which suggests syngamy failure. We hypothesize a role of Yem-alpha in remodeling the male pronucleus, a step that is required for syngamy [34]. This hypothesis is supported by two sets of published data. 1) On the one hand Yemanuclein-alpha/Ubinuclein/HPC2 define a new family of proteins found in HIRA chromatin remodeling complexes [17,35]; the HIRA WD domain interacting with a highly conserved domain of the Yem-alpha protein family [16]. 2) On the other hand mutations on the *Drosophila *HIRA protein prevent male pronucleus remodeling, precluding syngamy to occur [36]. This hypothesis is investigated elsewhere in collaboration with Loppin and coworkers (Orsi et al, in preparation).

In conclusion our work is the first to describe parthenogenetic development of adult flies as a result of a characterized mutation. More investigations are required to fully understand the underlying mechanisms.

### The chromosome segregation defects of yem^1 ^oocytes suggest kinetochore dysfunction

Acccording to Fuyama, the diploidy of the eggs produced by the *gyn-F9 *females is the result of the fusion of two of the polar bodies produced by an otherwise normal meiotic division [32,33]. Without further molecular or cytological data to support this view the interpretation advanced by Fuyama is possible but other mechanisms may not be ruled out. In contrast, the present work strongly supports the model that in *yem^1^/Df3450 *mutants, meiotic segregation defects key to the formation of diploid eggs.

The cytological analysis of *yem^1 ^*mutants in meiosis revealed abnormal meiotic figures consistent with defects in attachment of chromosomes to the meiosis I spindle. Moreover most of the recombination defective mutations partially restore fertility in *yem^1 ^*females but they do so differentially (Additional file 1, Table S3), pinpointing the importance of meiosis in the formation of the diploid eggs (that develop as parthenogenetic adult flies). Interestingly *mei-W68 *that fails to rescue the sterility shows a strong genetic interaction with *yem^1 ^*at early pachytene, which is not the case for *mei-218 *and *mei-9 *(Meyer et al, in preparation). Therefore early meiotic events might interfere with the formation of viable diploid eggs. Although we are not able to interpret these data, they underscore the importance of meiosis in their formation.

As the *yem^1 ^*induced phenotypes have no precedent in *Drosophila*, we searched for analogous meiotic phenotypes in other model organisms. In yeast, two mutations, *spo12 *and *spo13*, result in diploid spore formation [37]. However the underlying mechanisms are different. In *spo12 *mutants, the single division is essentially reductional [38] whereas in *spo13 *it is mostly equational. A recombination defective background has opposite effects on the two mutations with an increase of equational segregation in *spo13*, the phenotypes of which result from kinetochore defects [28,29]. The *spo13 *defect in yeast allows the attachment of sister kinetochores to microtubules from opposite spindle poles at meiosis I, and consequently, there is a delay of meiotic progression.

**Figure 6:**
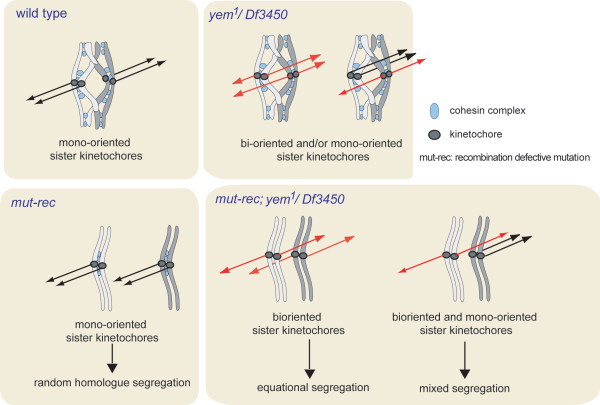
**Working model integrating the cytological observations and the genetic analysis of yem^1 ^mutants to account for the formation of the diploid eggs**. In a wild type oocyte chromatids arms of the homologues undergo exchange. The kinetochores of the sister chromatids whose orientation is monopolar are maintained by meiosis I specific cohesion. This configuration is necessary and sufficient for metaphase I arrest in the mature oocyte. In a recombination defective mutant (*mut-rec*), homologues are not maintained any longer through their arms: no tension is generated. Meiosis resumes prematurely in the oocyte before ovulation (precocious anaphase) and the homologues fail to segregate properly. These defects do not affect sister chromatids, neither their centromeric cohesion nor their kinetochores monopolar orientation. In an oocyte that is mutant for *yem-alpha *(*yem^1^/Df3450*), meiosis arrests at metaphase I. Defective orientation of sister kinetochores may occur to some extent (such as bi-orientation at meiosis I). In a recombination defective background, *yem^1^/Df3450 *oocytes resume meiosis prematurely. When bi-oriented sisters lose centromeric cohesion before undergoing poleward migration a single division occurs. If conditions are met (segregation of an appropriate set of chromosomes), viable diploid eggs may form with a single or two X chromosomes. The exceptional progeny being parthenogenetic they are essentially females (egg with 2 maternal X chromosomes). A fraction of sterile males developed from eggs with a single maternal X chromosome. This suggests the possibility that these eggs resulted from an equational division with some mixed segregation occurring too.

Thus examination of our data in light of these mutants strongly suggests the possibility that *yem^1 ^*mutation affects the kinetochores. First, *yem^1 ^*affects meiosis progression (as tested in the recombination defective backgrounds). Then, the frequency of the parthenogenetic offspring (that presumably reflects the frequency of the diploid eggs) is increased in a recombination defective context. These progeny bear the homologous chromosomes of the mother. Moreover Yem-alpha localizes to the kinetochore. Although full demonstration remains to be provided the formation of the diploid eggs can be accounted for by an equational division during *yem^1 ^*mutant meiosis. This is discussed in a tentative model (Figure 6).

We wanted to genetically address the effect of centromeric cohesion removal on *yem^1 ^*mutant oocytes by using a mutation on the *mei-S332 *Shugoshin homologue [39,40] that affects essentially meiosis II as a result of centromeric cohesion loss at late anaphase I [41]. As we failed to recover *mei-S332*; *yem^1 ^*double mutant individuals no conclusive experiment could be performed. Neither could we find exceptional progeny when we combined *yem^1 ^*with *ord*, a mutation that affects cohesion [42]. A gene required for centromeric cohesion in the male meiosis (SOLO) has recently been reported [43]. It will be interesting in the future to test whether Yem-alpha and SOLO interact for kinetochore function during female meiosis I [43]. Large-scale approaches have already been conducted to identify spindle and/or kinetochore functions in *Drosophila *mitosis [44-46]. Such endeavors might reveal more difficult for female meiosis.

### Yemanuclein-alpha function

The biochemical characterization of the Yemanuclein-alpha function is at its beginning. Only recently Yemanuclein-alpha and its orthologues in Yeast (HPC2) and Humans (UBN1) were shown to be part of the HIRA histone chaperone complex [15-17,35]. Interestingly connections between kinetochores and HIRA or Yem-alpha orthologues have been documented [15]. Hir, the HIRA yeast homologue was shown to contribute to building functional kinetochores [47,48]. In mammalian cells recruitment of hMis12 kinetochore protein is dependent on histone H3.3 deposition by HIRA [49,50], as is male pronucleus remodeling in Drosophila eggs [36]. Therefore, there is a strong connection between HIRA protein complexes (in which Yema-alpha and its orthologues were found), kinetochore function and male pronucleus remodeling. These molecular data, coupled with our genetic and cytological observations provide the basis for investigating Yemanuclein-alpha function in mediating the assembly of appropriate meiosis I specific kinetochores in oocytes and male pronucleus remodeling. The specificity acquired by Drosophila Yemanuclein-alpha for maternal functions is intriguing. A possible interpretation is that it might have been acquired from the adaptive use of the *Drosophila *protein for specific needs of sexual reproduction.

## Conclusions

This work raises new concepts and outstanding questions in the field of meiosis and sexual reproduction. Future investigations will be dedicated to understanding how these two faces of the same coin, namely diploidy reduction and diploidy restoration, are mechanistically linked and how they have evolved to maintain sexual reproduction.

## Methods

The identification and the molecular characterization of the *yem^1 ^*mutant allele are described as Additional Methods. Additional file 1, Table S4 summarizes the fly stocks used in the genetic experiments performed in this work.

### Immuno-staining of the spindles and the chromosomes

To prevent unwanted egg activation, the ovaries were manually dissected in cold methanol. Egg chorion and vitelline membrane were removed by sonication [51]. Immuno-staining procedures were performed as described elsewhere, using primary antibodies against tubulin 1/1000e (monoclonal T9026 Sigma) and DAPI. DNA was DAPI stained according to the supplier recommendation (Roche). Secondary antibodies were FITC-conjugated anti-mouse antibody (Jackson ImmunoResearch Labs). A Leica DMRA2 microscope was used for conventional epifluorescence.

As phospho-histone H3 cannot be detected in the conditions described above, we adapted a method that does not use methanol [4]. The rabbit histone H3 (phospho-Ser10) polyclonal antibody (Upstate) was used at 1/200. Secondary antibodies were FITC-conjugated anti-mouse antibody and TRITC-conjugated anti-rabbit antibody (Jackson ImmunoResearch Labs). Confocal microscopy was performed on a Zeiss LSM 410.

### Detection of Yem-alpha and CID on *whole mounts*

The Yemanuclein-alpha immunochemical staining was performed using the AS2 polyclonal antibodies from rabbits described in an earlier work [14]. The monoclonal anti-Orb antibody developed by [52] was obtained from the Developmental Studies Hybridoma Bank and used at 1:10000. CID staining was performed with a 1/100 chicken anti-CID antibody (G. Karpen) and revealed with a goat Cy2-coupled anti-chicken antibody (Abcam) at 1/250. To prevent loss of Yem-alpha antigenic response, we avoided any kind of specimen fixation. The procedure was as follows: the ovaries were incubated overnight at 4°C with the primary antibody after a blocking step in 1× PBS, 0.1% Triton and 5% low-fat dried milk. All the subsequent steps were performed in 1× PBS, 0.1% Triton and 0.5% low-fat dried milk.

### Detection of Yem-alpha and CID on oocyte frozen sections

The protocol used for immunochemical detection of Yem-alpha on frozen sections was described in detail in our previous publications [14,53]. Oocytes (stage 12 to 14) were hand-dissected, embedded in OCT (Gentaur) and frozen by immersion in liquid nitrogen before performing 7 μm thick serial sections. The treatment of the sections was as previously described. DNA was stained with DAPI (Roche). CID and Yem-alpha stainings were performed as indicated above.

### Genotyping of yem^1 ^exceptional progeny

Genomic DNA was prepared from single flies using classical procedures. A 582 bp fragment was amplified in a MJ Research MiniCycler using oligonucleotides OA71 and OA72. Sequencing reactions were performed with the DYEnamic ET Terminator Cycle Sequencing kit (Amersham Biosciences) using an internal oligonucleotide (OA69). Cycle sequencing reactions were run on a ABI PRISM 377 DNA Sequencer (Applied Biosystems).

OA71: 5'ACTCTGCTCCCCACCACATTG3'

OA72: 5'CAGTTCCACCACCTTTTCCTTGAG 3'

OA69: 5'GTACACATCGTACAGCAG3'

## Authors' contributions

OA conceived the study. OA, REM designed the experiments. REM, MC, OA performed the experiments. RR, MD, OA designed and/or performed genetic screens for female sterile mutations. OA, REM interpreted the data. OA wrote the manuscript. All authors read and approved the final manuscript.

## Supplementary Material

Additional file 1 **Additional file **1** contains Additional Methods, 4 Additional Tables and Additional References**.
Additional Methods
Genetic Methods
Genetic screens for yem-alpha mutant alleles
Rescue construct and germ line transformation
Sequencing of the yem-alpha mutant allele
Additional Tables
Table S1: Sequences used in the Clustal-W alignments
Table S2: Recombination defective genotypes used in this work (called mut-rec)
*Table S3: Fertility rate and X chromosome segregation for various maternal genotypes*.
Table S4: Drosophila stocks used in the work described in the main text
Additional References Click here for file
