# Supplementary material for: A single mutation results in diploid gamete formation and parthenogenesis in a Drosophila yemanuclein-alpha meiosis I defective mutant
Source: BMC Genet. 2010 Nov 16;11:104. doi: 10.1186/1471-2156-11-104 (PMC2998452; doi:10.1186/1471-2156-11-104)
Supplement: Additional file 1 — contains Additional Methods, 4 Additional Tables and Additional References. Additional Methods Genetic Methods Genetic screens for yem-alpha mutant alleles Rescue construct and germ line transformation Sequencing of the yem-alpha mutant allele Additional Tables Table S1: Sequences used in the Clustal-W alignments Table S2: Recombination defective genotypes used in this work (called mut-rec) Table S3: Fertility rate and X chromosome segregation for various maternal genotypes. Table S4: Drosophila stocks used in the work described in the main text Additional References [file 1471-2156-11-104-S1.PDF]

ADDITIONAL FILE

A single mutation results in diploid gamete formation and parthenogenesis in a *Drosophila yemanuclein-alpha* meiosis I defective mutant

Régis E. Meyer[1], Michèle Delaage[2], Roland Rosset[2], Michèle Capri[1, 2]
And Ounissa Aït-Ahmed [1, 2, 3] *

1 Institut de Génétique Humaine (IGH), Unité Propre de Recherche 1142, Centre National de la Recherche Scientifique (CNRS), 141 Rue de la Cardonille, 34396 Montpellier cedex 5, France

2 Previous address: IBDML, Campus de Luminy Case 907, 13288 Marseille, Cedex 09, France
3 Previous address: Department of Biological Sciences, Stanford University, Stanford, CA94305, USA

* Corresponding author: Ounissa.Ait-Ahmed@igh.cnrs.fr
Institut de Génétique Humaine (IGH), Unité Propre de Recherche 1142, Centre National de la Recherche Scientifique (CNRS), 141 Rue de la Cardonille, 34396 Montpellier cedex 5, France
Tel : (33) +4 99 61 99 11
Fax : (33) +4 99 61 99 01

Régis E. Meyer current address
Cell Cycle and Cancer Biology
Oklahoma Medical Research Foundation
825 N.E. 13th Street
Oklahoma City, Oklahoma 73104

Additional Methods

Genetic Methods

Genetic screens for yem-alpha mutant alleles

Large segmental aneuploidy in some *Drosophila* gene regions may result in haploinviability, therefore a method which allows the recovery of small synthetic deficiencies by combining a deficiency and a duplication with displaced autosomal breakpoints was designed (Lindsley et al., 1972). In this work we used a stock called *Df78/82* that combines *Df(3R)A82* and *Dp(3R)R78*. This combination results in a small segmental aneuploidy in 98F and we showed, using molecular and cytological methods, that such a stock bears a single copy of the *yem-alpha* locus. The males were *T(3;Y)R78/* Y^S*.X.*Y^L*, y; T(Y;3)A82/ TM6* and the females were *T(3;Y)R78/ X.X; T(Y;3)A82/ TM6* (Kongsuwan et al., 1986). To generate a more convenient stock, the males of this stock were X-ray irradiated. One stable event was recovered that linked *R78* duplication to the attached *X.Y, y* chromosome. The genotype of this new deficiency stock was called *Df(R23)* is Y^S*.X.*Y^L*, T(3;X.Y)R78, y/ X/ Y; T(Y;3)A82 / TM3,Sb Ser*. It is conveniently written as follows: *Dp(3;X.Y)R78, y/ X/ Y*; *Df(3R)A82/ TM3, Sb Ser*. We refer to this deficiency stock as *R23*.

R23 was used to screen for EMS induced female sterile mutations in the 98F interval. For the F1 cross, *red e/red e* males were EMS fed using a classical procedure and crossed to *e Pr ca/TM3, Sb Ser* females. F2 *red e/TM3, Sb Ser* males were crossed individually to *R23* females. F3 females to be used in the fertility test were scored against the TM3 markers. These non TM3 flies have necessarily received *Dp(3R)* and *Df(3R)* chromosomes and a mutagenized *red e** chromosome, they were then crossed to males from a wild type stock to assess female fertility. The *red e*/TM3* flies were kept to establish stocks whenever the *red e** chromosome confers sterility to the females. Altogether in this mutagenic screen, 2000 mutagenized chromosomes were tested for female sterility over *R23* deficiency. 4 female sterile mutations were recovered

and tested with a transgenic line that expresses ectopic *yem-alpha* under the control of its own promoter *(pyemA2).* One out of the 4 mutations could be rescued by the transgene. This mutation is the first one reported in the *yem-alpha* locus, therefore we call it yem^1.

As an interstitial deficiency recovered by S. Hayashi was made available for the 98F region, we performed a new EMS mutagenic screen for female sterile mutations in the interval covered by *Df(3R)3450* with the objective of identifying more *yem-alpha* mutant alleles. 7 female sterile mutations were obtained for 5000 chromosomes tested, none of them could be rescued by *pyemA2* line. P insertion mutagenesis was also performed but after numerous attempts, we failed to find any P element inserted within or nearby *yem-alpha* locus. We also screened the female sterile collection on the third chromosome provided by C. Nusslein-Volhard over *Df(3R)3450.* Two of the fs mutations were not complemented by the deficiency, but none was rescued by *yem-alpha* transgene. The original yem^1 chromosome was rid by recombination of a lethal mutation that was associated to a different locus. We first used yem^1 homozygous females for the analysis of the meiotic defects and then $yem^1/Df3450$ was used in most of the experiments after having verified that the phenotypes were consistent between the two genotypes.

Rescue construct and germ line transformation

The rescue transgene was constructed by inserting a *yem-alpha* EcoRI genomic fragment which contains all *yem-alpha* regulatory sequences including its promoter into pCasper 4 vector (Ait-Ahmed et al., 1992). Germ line transformation was as described earlier (Capri et al., 1997). The ability of this construct to rescue $yem^1/Df3450$, $yem^1/R23$, or yem^1/yem^1 female sterility was tested by using various transgenic lines bearing the transgene on the X or the 2nd chromosomes. All of them were able to rescue yem^1 phenotype.

Sequencing of the yem-alpha mutant allele

Overlapping DNA fragments were generated by PCR amplification of genomic DNA with appropriate *yem-alpha* oligonucleotides using a PROMEGA Taq and an MJ Research Minicycler^{TM}. Sequencing was performed by Genome Express. Both strands of *yem*^{1} allele were sequenced. The parental *red e* chromosome used in the mutagenic screen was also sequenced using the same strategy. This precaution is important to account for possible DNA polymorphism between the *Drosophila* stocks used in the present work and the stock from which *yem-alpha* sequence was established (Ait-Ahmed et al., 1992).

It is worth mentioning that very recently new genetic aberrations on *yem-alpha* have been reported and made publically available. They will be characterized in the future.

Additional Tables

Table S1 – Sequences used in the Clustal-W alignments

**Name**	**Species**	**Accession number**
Yem-α	*Drosophila melanogaster*	GenBank accession n° P25992
HsUbn1 (Ubinuclein)	*Homo sapiens*	GenBank accession n° NP_058632
HsUbn2	*Homo sapiens*	GenBank accession n° NP_775840
XlUbn2	*Xenopus laevis*	ESTs, assembled by TIGR as TC356543 (*http://www.tigr.org/tdb/tgi*)
MmUbn2	*Mus musculus*	GenBank accession n° NP_796159
MmUbn1	*Mus musculus*	GenBank accession n° NP_058632
VT4 cDNA	*Homo sapiens*	GenBank accession n° U19346

Yem-alpha sequence was reported long before its human similar sequence Ubinuclein (Aho et al., 2000; Ait-Ahmed et al., 1992). Yem-alpha and Ubn1 are the only sequences whose function has been experimentally addressed.

Table S2 - Recombination defective genotypes used in this work (called *mut-rec*)

Genotype	Homologue and/ or function	Effect on meiosis Progression	Reference
mei-9	Rad1 (Sc), XPF (Mam.)	Precocious anaphase	1, 2, 3
mei-W68	Spo11, Rec12 (Sp) DSB formation	Precocious anaphase	4, 5, 6
ord	Cohesin/ SC maintenance	Precocious anaphase	7
mei-218	CO formation	Precocious anaphase	1, 2
mei-217	CO formation	Precocious anaphase	8
c(3)G	SC component	Precocious anaphase	4, 9

Sc: *Saccharomyces cerevisiae*, Sp: *Schizosaccharomyces pombe*, Mam: Mammalian; CO: Cross Over, DSB: Double Strand Break, SC: Synaptonemal Complex.
1. (McKim et al., 1993), 2. (Carpenter and Sandler, 1974), 3. (Sekelsky et al., 1995), 4. (McKim et al., 2000), 5. (McKim and Hayashi-Hagihara, 1998), 6. (McKim et al., 1998), 7. (Bickel et al., 2002), 8. (Liu et al., 2000), 9. (Page and Hawley, 2001).

Table S3 - Fertility rate and X chromosome segregation for various maternal genotypes

male parent X chromosome Xp	female parent X chromosome Xm	male progeny X chromosome		female progeny X chromosome			
		fertile /sterile✪	sterile	fertile	fertile§	sterile	
	cross 1 Xm/Xm	Xm	Xp	XmXp	XmXm	XmXm	
	cross 2,3,4,5 Xm1/Xm2	Xm1 or Xm2	Xp	Xm1Xp or Xm2Xp		Xm1Xm2	Xm1Xm1 or Xm2Xm2

Genotype of the mother	nb mothers	fertility rate	nb progeny	cross	progeny number for each segregation class					
**wt**	40	10253	4101	1	1811	0	2290	0	0	
X/FM7	60	9637	5782	2	2645	23	3091	23	0	0
***mei-218*^{1}**	33	2224	734	1	295	53	321	65	0	
***c(3)G*^{68}**	40	7140	2856	1	1231	287	1109	229	0	
***mei-9*^{a}**	30	4663	1399	1	521	146	732		0	
ord*^{5}*/Df	69	519	358	1	115	55	111	77	0	
mei-9*^{a}*; yem*^{1}*/Df	**1615**	**2,79**	**45**	**5**	**1**	**0**	**7**	**0**	**37**	**0**
mei-217*^{g10}*; yem*^{1}*/Df	**526**	**14,26**	**75**	**1**	**27#**	**0**	**3**	**0**	**45**	
mei-218*$^{1/8}$*; yem*^{1}*/Df	**344**	**18,02**	**62**	**3**	**10**	**0**	**0**	**0**	**52**	**0**
***yem*^{1}*/Df* ***	**7474**	**1,14**	**85**	**4**	**26**	**0**	**28**	**0**	**25 ***	**6***
X/FM7; yem*^{1}*/Df	**3196**	**1,69**	**54**	**2**	**10**	**0**	**15**	**0**	**27**	**2**

Fertility rate was calculated as number (nb) of progeny for 100 mothers. Wild type was determined in our experimental conditions.
The progeny were scored for X chromosome segregation in four types of crosses.
Cross 1: Female *y/y* **X** Male *w*/Y; **Cross 2**: Female *yw/y w B* **X** Male *w*/Y
Cross 3: Female *y/y cv v f* **X** Male *w*/Y; **Cross 4**: Female *y/y w* **X** Male *w*/Y
Cross 5: Female *v/f* **X** Male *y w B*/Y
Crosses 2,3,4,5 allow to score the 2 maternal X as they bear different markers making it possible to infer the type of division. When no expression of the recessive markers is observed in the progeny, it means that they are Xm1/Xm2. For cross 3 analyzed in detail in Figure 5, no *y cv v f /y cv v f* progeny were recovered, we inferred that the female progeny were *y/y cv v f*. The stocks were not isogenized for the X chromosome but both chromosomes are viable as hemizygous in the males and the results are consistent in all these experiments with different X chromosomes.
§ These fertile females are normally XXY and developed from 2 Xm-eggs.
# Not tested individually, produced extremely rare offspring, essentially sterile as the female siblings.
* The X chromosome is here in a recombination proficient background. Therefore some of the sterile females* may have chromosomes that underwent exchange. This difficulty is alleviated with the cross that uses non exchange X chromosomes in the same genetic background. FM7 is an achiasmate balancer chromosome with *y, w* and *B* markers.
Light purple: sterile progeny (male only) resulting from nullo-X eggs (have paternal X markers).
Dark purple: sterile progeny (male and female) with no paternal X markers. These progeny were specifically recovered from females that are mutant for *yem-alpha*.
✪ These sterile males have maternal X markers, therefore they developed from Xm0 eggs. Can be distinguished from the XmY males only when the Y chromosome is marked (see Fig. 5D).

Table S4 - Drosophila stocks used in the work described in the main text

Strain	***Genotype***	***Origin***
OA69	*w[1118]*	Bloomington
OP53	*yem[1] red e/TM3, Sb e*	This study
OP55	*w[1118]; Df(3R)3450/TM3, Sb*	S. Hayashi
OA86	*y/y[+]Y; mei-W68[1]/CyO*	T. Schupbach
OA91	*mei-9[a] f[36]/C(1)DX, y f/y[+]Y*	T. Schupbach
RG15	*mei-9[a] f(36]/FM7a; spa[pol]*	This study
OA150	*mei-9[a] v/FM7; ry[531]*	KS. McKim
OA108	*yw mei-217[g10]/FM7c/y[+]Y*	KS. McKim
OA90	*y mei-218[1]/ C(1)DX, yf/y[+]Y; spa[pol]*	T. Schupbach
RG09	*y mei-218[1]/FM7a/y+ Y; spa[pol]*	This study
OA92	*y cv v mei-218[8] f/C(1)DX/y[+]Y*	T. Schupbach
OA99	*y[1]/Dp(1;Y)y[+]; Df(2R)3-70, cn[1]/CyO, bw*	SE. Bickel
OA100	*y[1]/Dp(1;Y)y[+]; ord[5] bw[2]/SM1; pol*	SE. Bickel
OA149	*y[1]/Dp(1;Y)y[+]; c(3)G[68] ca/TM3, Sb*	KS. McKim
RM05	*y mei-218[1]/FM7a; yem[1] red e/TM3,Sb e*	This study
RM06	*y mei-218[1]/FM7a; Df(3R)450/TM3,Sb e*	This study
RM07OA	*y cv v mei-218[8] f/FM7a; yem[1] red e/TM3,Ser e*	This study
RM07OASb	*y cv v mei-218[8] f/FM7a; yem[1] red e/TM3,Sb e*	This study
RM08	*y cv v mei-218[8] f/FM7a; Df(3R)3450/TM3,Ser e*	This study
RM09	*y; mei-W68[1]/CyO; yem[1] red e/TM3,Ser e*	This study
RM10	*w; mei-W68[1]/CyO; Df(3R)3450/TM3,Ser e*	This study
RM11	*yw mei-217[g10]/FM7a; yem[1] red e/TM3,Sb e*	This study
RM12	*yw mei-217[g10]/FM7a; Df(3R)3450/TM3,Sb e*	This study
RM13	*mei-9[a] v/FM7a; yem[1] red e/ TM3,Sb e*	This study
RM14	*mei-9[a] f[36]/FM7a; Df(3R)3450/TM3,Sb e*	This study
RM21	*Df(2R)3-70/CyO; yem[1] red e/TM3, Ser*	This study
RM22	*w; ord[5] bw[2]/CyO; Df(3R)3450/TM3, Ser e*	This study
RM29	*yw/FM7a; yem[1] red e/TM3,Sb e*	This study

The complete genotype is indicated in the text only when necessary. *Dp(1;Y)y[+]* is conveniently written *y[+] Y* or for more simplification, sometimes Y^{y+} in the text.

Additional References

Aho, S., Buisson, M., Pajunen, T., Ryoo, Y.W., Giot, J.F., Gruffat, H., Sergeant, A. and Uitto, J. (2000) Ubinuclein, a novel nuclear protein interacting with cellular and viral transcription factors. J Cell Biol 148, 1165-76.

Ait-Ahmed, O., Bellon, B., Capri, M., Joblet, C. and Thomas-Delaage, M. (1992) The yemanuclein-alpha: a new Drosophila DNA binding protein specific for the oocyte nucleus. Mech Dev 37, 69-80.

Bickel, S.E., Orr-Weaver, T.L. and Balicky, E.M. (2002) The sister-chromatid cohesion protein ORD is required for chiasma maintenance in Drosophila oocytes. Curr Biol 12, 925-9.

Capri, M., Santoni, M.J., Thomas-Delaage, M. and Ait-Ahmed, O. (1997) Implication of a 5' coding sequence in targeting maternal mRNA to the Drosophila oocyte. Mech Dev 68, 91-100.

Carpenter, A.T. and Sandler, L. (1974) On recombination-defective meiotic mutants in Drosophila melanogaster. Genetics 76, 453-75.

Kongsuwan, K., Dellavalle, R.P. and Merriam, J.R. (1986) Deficiency Analysis of the tip of chromosome 3R in *Drosophila melanogaster*. Genetics 112, 539-550.

Lindsley, D.L., Sandler, L., Baker, B.S., Carpenter, A.T., Denell, R.E., Hall, J.C., Jacobs, P.A., Miklos, G.L., Davis, B.K., Gethmann, R.C., Hardy, R.W., Steven, A.H., Miller, M., Nozawa, H., Parry, D.M. and Gould-Somero, M. (1972) Segmental aneuploidy and the genetic gross structure of the Drosophila genome. Genetics 71, 157-84.

Liu, H., Jang, J.K., Graham, J., Nycz, K. and McKim, K.S. (2000) Two genes required for meiotic recombination in Drosophila are expressed from a dicistronic message. Genetics 154, 1735-46.

McKim, K.S., Green-Marroquin, B.L., Sekelsky, J.J., Chin, G., Steinberg, C., Khodosh, R. and Hawley, R.S. (1998) Meiotic synapsis in the absence of recombination. Science 279, 876-8.

McKim, K.S. and Hayashi-Hagihara, A. (1998) mei-W68 in Drosophila melanogaster encodes a Spo11 homolog: evidence that the mechanism for initiating meiotic recombination is conserved. Genes Dev 12, 2932-42.

McKim, K.S., Jang, J.K., Sekelsky, J.J., Laurencon, A. and Hawley, R.S. (2000) mei-41 is required for precocious anaphase in Drosophila females. Chromosoma 109, 44-9.

McKim, K.S., Jang, J.K., Theurkauf, W.E. and Hawley, R.S. (1993) Mechanical basis of meiotic metaphase arrest. Nature 362, 364-6.

Page, S.L. and Hawley, R.S. (2001) c(3)G encodes a Drosophila synaptonemal complex protein. Genes Dev 15, 3130-43.

Sekelsky, J.J., McKim, K.S., Chin, G.M. and Hawley, R.S. (1995) The Drosophila meiotic recombination gene mei-9 encodes a homologue of the yeast excision repair protein Rad1. Genetics 141, 619-27.
